# Comparison of sexual risk behaviors among Zambian adolescent girls and young women living with and without HIV

**DOI:** 10.1186/s12978-025-02147-2

**Published:** 2025-09-30

**Authors:** Suzannah L. Scanlon, Natalie A. Blackburn, Jenny Beizer, Drosin M. Mulenga, Laura Nyblade, Sarah T. Roberts, Nachela Chelwa, Michael Mbizvo, Sujha Subramanian

**Affiliations:** 1 RTI International, 5921 Monticello Rd, Alexandria, VA 22303 USA; 2 RTI International, 3040 E. Cornwallis Rd., P.O. Box 12194, Research Triangle Park, Durham, NC 27709 USA; 3 Population Council, 8 Nyerere Rd, Lusaka, Zambia; 4 Implenomics, 8 The Green, Suite #6172, Dover, DE 19901 USA

**Keywords:** Zambia, HIV, Sexual behavior, Contraception, Adolescent girls and young women

## Abstract

**Background:**

We sought to identify commonalities and variations in sexual risk behaviors between adolescent girls and young women living with and without human immunodeficiency virus (HIV) in Zambia. Our goal was to understand the specific needs of these populations to inform the design of interventions to support the sexual health by age group and HIV status.

**Methods:**

As a part of baseline survey Data collected for a cluster-randomized controlled, we surveyed a sample of 650 women aged 16–22 residing in Lusaka, Zambia between May and September 2021. We used bivariate statistical methods to determine whether sexual behavior and contraceptive use differed among participants living with and without HIV, by age group. Statistical significance was defined at *P* ≤ 0.10.

**Results:**

We found that among the younger participants (aged 16–18), those living with HIV were less likely to have ever had sex or be currently sexually active, and reported fewer casual and serious sexual partners in the last three months compared to those living without HIV. Among participants aged 19–22, we did not see a difference in sexual debut or number of casual sexual partners by HIV status. However, those living with HIV reported fewer recent serious sexual partners and were less likely to be currently having sex in this older age group. There was also evidence that those living with HIV in this older age group were more likely to be using condoms with sexual partners than their counterparts without HIV.

**Conclusions:**

Future HIV interventions should be tailored by age group and HIV status. For example, those living with HIV may require support to confidently engage in safe sexual relationships.

**Trial registration:**

This study was approved by both U.S. and Zambian Institutional Review Boards (RTI Institutional Review Board and RES Converge Zambia, respectively) and is registered on clinicaltrials.gov (NCT03995953).

**Supplementary Information:**

The online version contains supplementary material available at 10.1186/s12978-025-02147-2.

## Abstract (plain language summary)

This study found differences in sexual behavior and use of contraceptives between teen girls and young women in Zambia, living with and without HIV. We surveyed 650 women ages 16–22 residing in Lusaka, the capital of Zambia. Among participants ages 16–18, we found that those living with HIV began having sex at an older age than those living without HIV. Those living with HIV were engaging in less current sexual activity than those living without HIV. This difference was not as stark among older participants, ages 19–22. Also, in this older age group (19–22), we found evidence that those living with HIV were more likely to be using condoms with their sexual partners. It was difficult to tell if this was the case in the younger age group of participants ages 16–18, since so few of the participants living with HIV in this age group reported having sex. These findings will aid in the design of youth-friendly sexual and reproductive health programs by providing information on how the behavior of teen girls and young women may be similar or different based on their HIV status and age.

**Supplementary Information:**

The online version contains supplementary material available at 10.1186/s12978-025-02147-2.

## Background

The risk of human immunodeficiency virus (HIV) acquisition and prevalence remains high among adolescent girls and young women (AGYW) in sub-Saharan Africa (SSA) [1–3]. Zambia has one of the highest incidences of HIV in the world due to both perinatal and behavioral infections, with AGYW particularly affected [4, 5]. Approximately 14 000 new infections occur annually among AGYW aged 15–24, and about 5% of girls aged 15–19 and 11% of young women aged 20–24 in Zambia are living with HIV [4, 6, 7].

Sexual behaviors that increase the risk of acquisition and transmission of HIV and other sexually transmitted infections (STIs) and of unintended pregnancy [8] include having a high number of sexual partners and not using condoms or other contraceptives [9]. An individual’s HIV status and knowledge of their HIV status may impact their likelihood of engaging in sexual risk behaviors. Understanding whether and how these behaviors differ between individuals living with and without HIV is important in designing interventions to prevent the acquisition and transmission of HIV and other negative health outcomes.

Several studies have found decreases in sexual risk behaviors after participants were notified that they were HIV positive [10–14]. Two of these studies took place in the United States [11, 12], one in China [13], and one [14] in Uganda. Another [10] was a systematic review and meta-analysis with studies from around the world. The meta-analysis portion of this study only contained one study from Zambia, which was specifically about female sex workers. A few studies have compared sexual risk behaviors between those living with and without HIV. One study of older adults in rural South Africa found that condom use was lower among adults living without HIV than among adults living with HIV who were aware of their status [15]. However, a study of adolescent sexual behavior in Nigeria found that HIV status was not associated with use of condoms [16]. This study in Nigeria was the only one we identified that focused on differences in sexual behavior between those living with and without HIV specifically among adolescents in SSA.

To our knowledge, no research has analyzed the differences in sexual behavior between AGYW living with and without HIV in Zambia. We sought to fill this gap by analyzing whether sexual behaviors and contraceptive use differ between AGYW living with and without HIV in Zambia’s capital, Lusaka. We analyzed self-reported survey data on sexual risk behaviors, comparing these behaviors between those living with and without HIV stratified by age group (16–18 and 19–22). This study will identify similarities and differences in sexual risk behaviors between AGYW in different age groups to better understand the specific needs of these populations and to aid in the design of interventions that address unique sexual health needs that may exist by age group and HIV status.

## Methods

Data for this study come from the baseline survey assessment of the Support for HIV Integrated Education, Linkages to Care, and Destigmatization and Integrated Wellness Care (SHIELD + IWC) program, a cluster-randomized controlled trial testing integrated care delivery for HIV prevention and treatment among AGYW in Zambia (herein referred to as the parent study).

### Recruitment of participants

We selected 6 distinct clinic zones in the greater Lusaka area that were not contiguous to recruit AGYW living with and without HIV. The parent study protocol has been previously published, which includes additional information on methods of recruitment, screening, informed consent, and data collection from study participants [17]. We provide a brief summary of the recruitment approach below.

#### Sampling technique

Individuals living without HIV were identified from the community and those living with HIV were identified from health facilities. To recruit the participants living without HIV, we mapped households around the study health facilities to identify participants. Study data collectors went household to household, beginning with residential areas closest to the clinics to identify those potentially eligible based on inclusion and exclusion criteria. To recruit participants living with HIV, we requested that health clinic staff identify eligible AGYW through heath facility records. The clinic staff then contacted the AGYW to seek their consent to be contacted by the research team about the study to maintain confidentiality. The study data collectors reached out to those who pre-consented for the study.

#### Inclusion and exclusion criteria

For participants living without HIV, participant had to be female, 10–20 years of age, not pregnant, and self-report their HIV status as negative or unknown (“unknown” defined as no HIV testing within the past 6 months). For participants living with HIV, participants who were female, 16–24 years of age, not pregnant, and diagnosed with HIV within the past three years (to target relatively recently diagnosed AGYW at the time of recruitment) were eligible.

### Study participants and survey measures

Baseline survey Data were collected from participants from May through September 2021. Participants in this study were a subset of 650 participants from the parent study who self-reported their age to be between 16 and 22. We selected participants 16 and older because we did not ask questions about sexual behavior to those younger than 16 since sexual intercourse with those younger than 16 is criminalized in Zambia. The data we analyzed came from the baseline survey assessment administered to participants before the parent study interventions began.

The survey questions about sexual behavior analyzed in this study are common data elements recommended by the Prevention and Treatment through a Comprehensive Care Continuum for HIV-Affected Adolescents in Resource Constrained Settings (PATC^3^H) consortium [18], with some alterations made to fit the study context and setting. The survey questions about contraceptive use were developed though our own formative research [17, 19, 20]. The survey questions analyzed in this study are included in Additional File 1. Questions 1–6 were recommended by the PATC^3^H consortium and 7–10 were developed though our research.

### Data collection and management

Trained data collectors administered the baseline survey in person with participants in a private location, typically the participant’s home. They collected data on a tablet using REDCap's Mobile App [20], which allowed for secure, offline data capture and subsequent upload to a secure REDCap server. Interviews were conducted in Bemba, Nyanja, or English languages based on participant preference. For the portion of the survey focusing on sexual behavior, participants could have the data collector administer the questions or self-administer that section of the survey.

Following data collection, the dataset was reviewed for completeness, consistency, and accuracy. Data cleaning involved checking for logical inconsistencies, such as contradictory responses within skip patterns, and excluding responses that were missing due to skip logic or marked as “decline to answer.” Personally identifiable information was not included in the analytic dataset, and all data were de-identified prior to analysis.

### Variables

In this study, the independent variable was HIV status (living with HIV vs. not living with HIV), as self-reported by participants. The dependent variables were the responses to the baseline survey questions related to sexual behavior and contraceptive use.

### Analysis

We conducted bivariate analyses to determine whether sexual behavior and contraceptive use differed among participants living with and without HIV. Pearson Chi-Square tests were used to test the significance of association between HIV status and categorical or ordinal variables. T-tests were used to test the significance of association between HIV status and continuous variables. Analysis of data was performed in Stata (v. 17.0). Statistical significance was defined at *P* ≤ 0.10. We chose *P* ≤ 0.10 due to limited sample size and the exploratory nature of this investigation.

When performing bivariate tests, participants were stratified into younger (aged 16–18) and older (aged 19–22) age groups because sexual behavior is expected to change as people age from adolescents to young adults [21–23]. Ages 16–18 and 19–22 were selected for stratification based on our findings that specific changes indicative of transitioning from adolescence to adulthood occurred between the ages of 18 and 19 within our study population. Between these ages, we saw a marked decrease in the proportion of participants currently enrolled in school and an increase in the proportion of participants who had children and who were currently employed.

When performing bivariate tests, responses of *decline to answer* were treated as missing. In certain instances, responses of participants who were not asked a question due to a skip pattern in the survey were also treated as missing. Questions about sexual behavior were only analyzed among those who reported having had sex. Questions about current contraceptive use were only analyzed among participants who were determined to be sexually active and not trying to get pregnant at the time of the survey.

### Operational definitions

#### Currently sexually active and not trying to get pregnant

There was no question in the survey directly asking participants whether they were currently having sex or trying to get pregnant. For the purposes of analysis, we considered participants to be sexually active and not trying to get pregnant at the time of the survey if (1) they reported at least one sexual partner in the last three months, and (2) they did not list ‘not currently having sex’ or ‘trying to get pregnant’ as reasons for not currently using contraception.

#### Currently sexually active

Only three participants in our sample were determined to be trying to get pregnant; so for simplicity, the previously defined subset of participants determined to be currently sexually active and not trying to get pregnant is hereafter referred to as those currently sexually active.

#### Casual partner

A casual partner was defined as someone the respondent had sex with occasionally or one time.

#### Serious partner

A serious partner was defined as someone with whom the respondent had an ongoing relationship, like a lover, boyfriend, or someone they dated and felt very close to.

## Results

### Demographics and HIV Acquisition

Among the 650 AGYW sampled, 366 (56.3%) were living with HIV and knew their status (herein referred to as living with HIV), and 284 (43.7%) were living without HIV or did not know their status (herein referred to as living without HIV). The average age among participants was 19; however, due to the parent study design, the average age among participants living with HIV was older (20) than the average age (18) among those living without HIV. Most participants were single and did not have children. Table 1 displays demographic data on the AGYW participating in this study.

**Table 1:** Characteristics of AGYW Living With and Without HIV in Zambia, 2021 (*n* = 650)

	**Ages 16–18**		**Ages 19–22**		**Total**
	**Living with HIV n, (%)**	**Living without HIV n, (%)**	**Living with HIV n, (%)**	**Living without HIV n, (%)**	
Total	108, (100.0)	186, (100.0)	258, (100.0)	98, (100.0)	650, (100.0)
Level of Education^a^					
None	0, (0.0)	3, (1.6)	6, (2.3)	1, (1.0)	10, (1.5)
Primary School^b^	23, (21.3)	55, (29.6)	50, (19.4)	29, (29.6)	157, (24.2)
Secondary School^b^	82, (75.9)	128, (68.8)	187, (72.5)	66, (67.3)	463, (71.2)
College^b^	3, (2.8)	0, (0.0)	14, (5.4)	1, (1.0)	18, (2.8)
Currently in School	72, (66.7)	120, (64.5)	62, (24.0)	20, (20.4)	274, (42.2)
Relationship Status^a^					
Single	105, (97.2)	185, (99.5)	225, (87.2)	77, (78.6)	592, (91.1)
Married	3, (2.8)	0, (0.0)	28, (10.9)	20, (20.4)	51, (7.8)
Divorced	0, (0.0)	1, (0.5)	4, (1.6)	1, (1.0)	6, (0.9)
Employed	2, (1.9)	14, (7.5)	48, (18.6)	18, (18.4)	82, (12.6)
Has a Child/Children	2, (1.9)	11, (5.9)	91, (35.3)	38, (38.8)	142, (21.8)
HIV Acquisition from^a^					
Mother	87, (80.6)	-	144, (55.8)	-	231, (63.1)^c^
Sex	1, (0.9)	-	65, (25.2)	-	66, (18.0)^c^
Unknown	17, (15.7)	-	47, (18.2)	-	64, (17.5)^c^
Other	3, (1.9)	-	1, (0.4)	-	3, (0.8)^c^
Age learned living with HIV^a^					
≤ 5	3, (2.8)	-	6, (2.3)	-	9, (2.5)^c^
6–10	26, (24.1)	-	46, (17.8)	-	72, (19.7)^c^
11–15	64, (59.3)	-	87, (33.7)	-	151, (41.3)^c^
16–18	13, (12.0)	-	59, (22.9)	-	72, (19.7)^c^
19–22	0, (0.0)	-	59, (22.9)	-	59, (16.1)^c^
On ART	108, (100.0)	-	256, (99.2)	-	364, (99.5)^c^
On PrEP					
Ever	-	2, (1.1)	-	4, (4.1)	6, (2.1)^d^
Currently	-	1, (0.5)	-	0, (0.0)	1, (0.4)^d^
Tested for HIV in last 6 months	-	4, (2.2)	-	13, (13.3)	17, (6.0)^d^

*ART* Antiretroviral therapy, *PrEP* Pre-exposure prophylaxis

^a^Not all participants answered this question, so counts and percentages will not sum to total

^b^Primary school, secondary school, and college include those who have completed at least some school at this level

^c^Among those living with HIV, *n* = 366

^d^Among those living without HIV, *n* = 284

Among those living with HIV, the average age that participants reported being aware that they were HIV positive was 14. In the 16–18 age group, 80.6% reported they acquired HIV from their mother at birth or through breastfeeding, compared to 55.8% in the 19–22 age group. Conversely, less than 1% of those in the 16–18 age group reported acquiring HIV through sex, compared to 25.2% of those in the older age group, *X*^*2*^ (4, *N* = 366) = 34.91, *p* = 0.000.

### Sexual Behavior and contraceptive use by HIV status and age group

#### Ever had sex and currently having sex

**Fig. 1:**
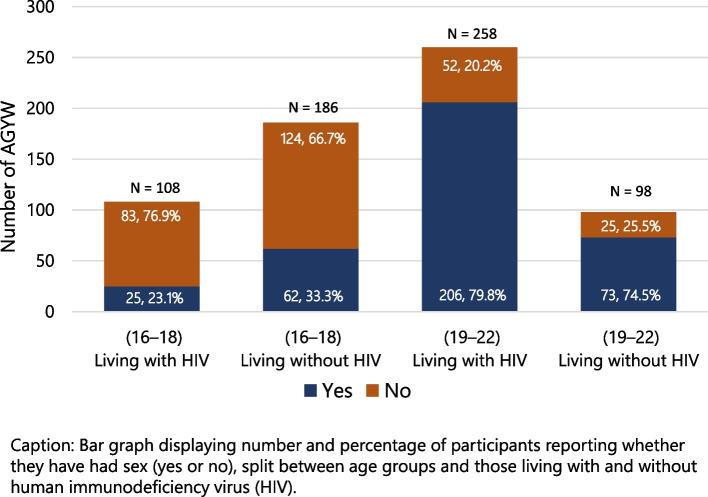
Participants reporting that they have had sex, by HIV status and age group. Caption: Bar graph displaying number and percentage of participants reporting whether they have had sex (yes or no), split between age groups and those living with and without human immunodeficiency virus (HIV). Values are displayed as n, %

In the 16–18 age group, the proportion of participants who reported ever having sex was lower among participants living with HIV (23.1%) than among participants living without HIV (33.3%), *X*^*2*^ (1, N = 294) = 3.40, *p* = 0.065. Conversely, in the 19–22 age group, a higher proportion of those living with HIV reported having had sex (79.8%) compared to those living without HIV (74.5%); however the difference was not statistically significant, *X*^*2*^ (1, *N* = 356) = 1.20, *p* = 0.273. Figure 1 displays the proportion of participants reporting whether they have had sex by HIV status and age group.

#### Age of sexual debut and number of sexual partners

Table 2 displays information on sexual partners among those who reported ever having sex. Among those who had ever had sex in both age groups, those living with HIV were less likely to be currently sexually active than those living without HIV. In the 16–18 age group, 32.0% of those living with HIV were currently sexually active, compared to 62.9% of those living without HIV, X^2^ (1, *N* = 87) = 6.85, *p* = 0.009. In the 19–22 age group, 63.6% of those living with HIV were currently sexually active, compared to 78.1% of those living without HIV, X^2^ (1, *N* = 279) = 5.15, *p* = 0.023.

**Table 2:** Sexual Partners Among AGYW in Zambia Who Have Had Sex, 2021 (*n* = 366)

	**Ages 16–18**			**Ages 19–22**			**Total**
	**Living With HIV**	**Living Without HIV**	**Statistic, Significance**	**Living With HIV**	**Living Without HIV**	**Statistic, Significance**	
Total: n, (%)	25, (100.0)	62, (100.0)	n/a	206, (100.0)	73, (100.0)	n/a	366, (100.0)
Currently sexually active: n, (%)	8, (32.0)	39, (62.9)	*X*^*2*^ (1, N = 87) = 6.85, *p* =.009	131, (63.6)	57, (78.1)	*X*^*2*^ (1, N = 279) = 5.15, *p* =.023	235, (64.2)
Age first had sex (mean, median, ± SD)	16.9, 17, ± 0.7	16.2, 16, ± 1.3	*t*(83) = − 2.43, *p* =.017	17.6, 18, ± 1.9	17.5, 18, ± 1.6	*t*(272) = − 0.31, *p* =.757	17.3, 17, ± 1.8
Number of sexual partners in lifetime (mean, median, ± SD)^a^	1.7, 1, ± 1.9	1.6, 1, ± 1.0	*t*(83) = − 0.30, *p* =.768	2.7, 2, ± 3.8	2.0, 2, ± 1.4	*t*(274) = − 1.60, *p* =.111	2.3, 2, ± 3.1
Number of sexual partners in lifetime: n, (%)^a^							
1–2	22, (88.0)	51, (82.3)	n/a	126, (61.2)	57, (78.1)	n/a	256, (69.9)
3–4	1, (4.0)	9, (14.5)		51, (24.8)	13, (17.8)		74, (20.2)
≥ 5	1, (4.0)	1, (1.6)		26, (12.6)	3, (4.1)		31, (8.5)
Number of casual partners in last 3 months (mean, median, ± SD)^a^	0.13, 0, ± 0.34	0.34, 0, ± 0.51	*t*(81) = 1.88, *p* =.063	0.52, 0, ± 0.93	0.47, 0, ± 0.63	t(274) = − 0.44, p =.658	0.47, 0, ± 0.79
Number of casual partners in last 3 months: n, (%)^a^							
0	21, (84.0)	40, (64.5)	n/a	124, (60.2)	42, (57.5)	n/a	227, (62.0)
1	3, (12.0)	18, (29.0)		67, (32.5)	27, (37.0)		115, (31.4)
≥ 2	0, (0.0)	1, (1.6)		13, (6.3)	3, (4.1)		17, (4.6)
Number of serious partners in last 3 months (mean, median, ± SD)^a^	0.54, 1, ± 0.51	0.81, 1, ± 0.60	*t*(84) = −1.92, *p* =.058	0.76, 1, ± 0.58	0.92, 1, ± 0.50	*t*(272) = 2.08, *p* =.039	0.78, 1, ± 0.57
Number of serious partners in last 3 months: n, (%)^a^							
0	11, (44.0)	17, (27.4)	n/a	61, (29.6)	11, (15.1)	n/a	100, (27.3)
1	13, (52.0)	41, (66.1)		132 (64.1)	57, (78.1)		243, (66.4)
≥ 2	0, (0.0)	4, (6.5)		9, (4.4)	4, (5.5)		17, (4.6)

^a^Not all participants answered this question, so counts and percentages will not sum to total

In the 16–18 age group, the average age of first sex was older for participants living with HIV (*M* = 16.9, *SD* = 0.7) than for participants living without HIV (*M* = 16.2, *SD* = 1.3), *t*(83) = − 2.43, *p* = 0.017. In the 19–22 group, average age of first sex was similar for both groups of participants.

The number of lifetime sexual partners was similar in the 16–18 age group. In the 19–22 age group, those living with HIV on average reported a greater number of sexual partners (*M* = 2.7, *SD* = 3.8) than those living without HIV (*M* = 2.0, *SD* = 1.4), however the difference was not statistically significant, *t*(274) = −1.60, *p* = 0.111. In the 16–18 age group, participants living with HIV reported fewer casual partners in the last three months (*M* = 0.13, *SD* = 0.34) than participants living without HIV (*M* = 0.34, *SD* = 0.51), *t*(81) = 1.88, *p* = 0.063. Participants living with HIV in this age group also reported fewer serious/main partners in the last three months (*M* = 0.54, *SD* = 0.51) than participants living without HIV (*M* = 0.81, *SD* = 0.60), *t*(84) = −1.92, *p* = 0.058.

In the 19–22 age group, participants living with and without HIV reported a similar number of casual partners in the last three months (M = 0.52 compared to M = 0.47, respectively). However, participants living with HIV reported fewer serious/main partners within the last three months (*M* = 0.76, *SD* = 0.58) than participants living without HIV (*M* = 0.92, *SD* = 0.50), *t*(272) = 2.08, *p* = 0.039.

#### Condom and other contraceptive use

Table 3 displays past contraceptive use among those who reported ever having sex. Most participants reported having used contraception (e.g., condoms, oral contraceptive pills, injectables, implants). While a higher proportion of those living with HIV in the 16–18 age group reported having used contraception (88.0%) compared to those not living with HIV (75.8%), the difference was not statistically significant. The proportion was similar between those living with and without HIV in the older age group (83.5% and 82.2%, respectively). Condoms were the most reported method of contraception across all groups. In both age groups, a higher proportion of those living with HIV reported ever having used condoms. While 80,0% of those living with HIV in the 16–18 age group reported having used condoms compared to 69.4% of those living without HIV, this difference was not statistically significant. The difference was statistically significant in the 19–22 age group, with 72.3% of those living with HIV reporting having used condoms compared to 47.9% of those living without HIV, *X*^2^ (1, *N* = 279) = 14.72, *p* < 0.001.

**Table 3:** Contraceptive Use Among AGYW in Zambia Who Have Had Sex, 2021 (*n* = 366)

	**Ages 16–18**			**Ages 19–22**			**Total**
	**Living With HIV n, (%)**	**Living Without HIV n, (%)**	**Statistic, Significance**	**Living With HIV n, (%)**	**Living Without HIV n, (%)**	**Statistic, Significance**	
Total	25, (100.0)	62, (100.0)	n/a	206, (100.0)	73, (100.0)	n/a	366, (100.0)
Overall Use of Contraception							
Ever used contraception	22, (88.0)	47, (75.8)	*X*^2^ (1, *N* = 87) = 1.61, *p* =.204	172, (83.5)	60, (82.2)	*X*^2^ (1, *N* = 279) = 0.07, *p* =.798	301, (82.2)
Condom Use							
Ever used condoms	20, (80.0)	43, (69.4)	*X*^2^ (1, *N* = 87) = 1.01, *p* =.315	149, (72.3)	35, (47.9)	*X*^2^ (1, *N* = 279) = 14.72, *p* <.001	247, (67.5)
Frequency of condom use with casual partners last 3 months^a^							
Never	1, (33.3)	4, (21.1)	*X*^2^ (2, *N* = 22) = 2.47, *p* =.291	24, (30.0)	11, (36.7)	*X*^2^ (2, *N* = 109) = 1.00, *p* =.605	40, (30.3)
Sometimes^b^	0, (0.0)	9, (47.4)		26, (32.5)	11, (36.7)		46, (34.8)
Always	2, (66.7)	6, (31.6)		29, (36.3)	8, (26.7)		45, (34.1)
Frequency of condom use with serious partners last 3 months^c^							
Never	2, (15.4)	10, (22.2)	*X*^2^ (2, *N* = 58) = 6.76, *p* =.034	38, (27.0)	27, (44.3)	*X*^2^ (2, *N* = 201) = 7.40, *p* =.025	77, (29.6)
Sometimes^b^	1, (7.7)	18, (40.0)		49, (34.8)	21, (34.4)		89, (34.2)
Always	10, (76.9)	17, (37.8)		53, (37.6)	13, (21.3)		93, (35.8)
Use of contraception other than condoms							
Ever used a contraceptive other than condoms (including oral contraceptive pills, injectables, and implants)	3, (12.0)	7, (11.3)	*X*^2^ (1, *N* = 87) = 0.01, *p* =.925	66, (32.0)	35, (47.9)	*X*^2^ (1, *N* = 279) = 5.91, *p* =.015	111, (30.3)
Discontinued Use of Contraception							
Not currently using contraception, but have in the past	14, (56.0)	15, (24.2)	n/a	62, (30.1)	18, (24.7)	n/a	109, (29.8)
Not currently using contraception because^d^							
Not currently having sex	13, (92.9)	12, (80.0)	n/a	47, (75.8)	9, (50.0)	n/a	81, (74.3)
Trying to get pregnant	0, (0.0)	0, (0.0)	n/a	3, (4.8)	0, (0.0)	n/a	3, (2.8)
Other reason (eg, worried about side effects or access issues)	1, (7.1)	3, (20.0)	n/a	11, (17.7)	9, (50.0)	n/a	24, (22.0)

^a^Among those with at least 1 casual partner (*n* = 132). Not all participants answered this question, so counts and percentages will not sum to total

^b^This is a combination of the responses “Less than half the time,” “About half the time,” and “More than half the time.” This combination of those 3 responses was used in bivariate tests

^c^Among those with at least 1 serious partner (*n* = 260). Not all participants answered this question, so counts and percentages will not sum to total

^d^Among those not currently using contraception, but who have in the past (*n* = 109). Not all participants answered this question, so counts and percentages will not sum to total

In the 16–18 age group, there were too few participants living with HIV that had at least one casual partner in the last three months (three participants) to compare condom use with those partners between those living with and without HIV. In the 19–22 age group, a higher proportion of those living with HIV reported always using condoms with casual partners (36.3%) compared to those living without HIV (26.7%), however the difference in condom use was not statistically significant. Within both age groups, a higher proportion of those living with HIV reported that they always used condoms with serious/main partners. In the 16–18 age group, 76.9% of AGYW living with HIV that had at least one serious partner in the last three months reported consistent condom use, compared to 37.8% of those living without HIV, *X*^2^ (2, *N* = 58) = 6.76, *p* = 0.034. In the 19–22 age group, 37.6% of those living with HIV reported consistent condom use compared to 21.3% of those living without HIV, *X*^2^ (2, *N* = 201) = 7.40, *p* = 0.025.

Table 4 displays information on current contraceptive use among those currently sexually active. Because only eight participants in the 16–18 age group living with HIV were currently sexually active, comparing current contraceptive use between those living with and without HIV in this age group lacks statistical power. The proportion of those currently using condoms in the older age group was higher among those living with HIV (55.0%) than those living without HIV (33.3%), *X*^2^ (1, *N* = 188) = 7.44, *p* = 0.006.

**Table 4:** Contraceptive Use Among Sexually Active AGYW in Zambia, 2021 (*n* = 235)

	**Ages 16–18**			**Ages 19–22**			**Total**
	**Living With HIV n, (%)**	**Living Without HIV n, (%)**	**Statistic, Significance**	**Living With HIV n, (%)**	**Living Without HIV n, (%)**	**Statistic, Significance**	
Total (Sexually Active)	8, (100.0)	39, (100.0)	n/a	131, (100.0)	57, (100.0)	n/a	235, (100.0)
Overall Use of Contraception							
Currently using contraception^a^	6, (75.0)	28, (71.8)	*X*^2^ (1, *N* = 47) = 0.03, *p* =.854	100, (76.3)	37, (64.9)	*X*^2^ (1, *N* = 188) = 2.62, *p* =.105	171, (72.8)
Condom Use							
Currently using condoms	4, (50.0)	26, (66.7)	*X*^2^ (1, *N* = 47) = 0.80, *p* =.371	72, (55.0)	19, (33.3)	*X*^2^ (1, *N* = 188) = 7.44, *p* =.006	121, (51.5)
Use of Contraception Other Than Condoms							
Currently using a contraceptive other than condoms	2, (25.0)	3, (7.7)	*X*^2^ (1, *N* = 47) = 2.09, *p* =.148	33, (25.2)	18, (31.6)	*X*^2^ (1, *N* = 188) = 0.82, *p* =.365	56, (23.8)

^a^Not all participants answered this question

## Discussion

We sought to determine whether sexual behaviors and contraceptive use differ between AGYW living with and without HIV in Lusaka, Zambia. Within the 16–18 age group, we found delayed sexual debut and less current sexual activity among those living with than those living without HIV. This difference in sexual activity by HIV status in the younger age group was not as stark in the older 19–22 age group, although those living with HIV in this age group reported fewer recent serious sexual partners and were less likely to be currently having sex. There was also evidence that those living with HIV in this age group were more likely to be using condoms with their past and current sexual partners than those living without HIV.

There is limited literature comparing sexual debut among AGYW living with and without HIV in SSA, but one 2014 study in Nigeria found no difference in age of sexual debut by HIV status in its sample of women aged 10–19 [16]. The study found the average age of sexual debut to be 14.8 among those living with HIV and 15.2 among those living without HIV, which was younger than we found in our sample of AGYW aged 16–18 [16]. The Zambian [24] Demographic and Health Survey (DHS) from 2018 found that the median age of first sexual intercourse among women aged 25–49 was 17.5 in Lusaka and that by age 18, 69% of women aged 20–24 in Zambia had had sexual intercourse. These Data on sexual debut are similar to what we found in the 19–22 age group; however, there were no Data available in the DHS collected from women younger than 19 that were comparable to our Data. Furthermore, a 2013 [25] study in Zambia found that 21% of adolescents (male and female) aged 15–18 who were living with HIV reported ever having sex, which is similar to our participants living with HIV in the 16–18 age group. Another 2013 study in the United States [26] found 53% of 16-year-old adolescents (male and female) with perinatally-acquired HIV had had sex, and that 67% of 18-year-olds with perinatally-acquired HIV had had sex.

The delayed sexual debut and less recent sexual activity among those living with HIV in the younger age group of our study may be rooted in the mode of HIV acquisition, which changes as AGYW mature. Almost no participants living with HIV in the younger age group reported acquiring HIV through sex, compared to about a quarter of participants living with HIV in the older age group. Other studies have shown that adolescents are more likely to have acquired HIV perinatally, but as adolescents age into young adults, a higher proportion acquire HIV through sex [27, 28]. The 2013 [25] study in Zambia found that 77% of adolescents (male and female) aged 15–18 reported acquiring HIV from their parents and only 4% from sex, similar to our sample. A 2010 [29] study in the United States found that being currently sexually active and having unprotected sex was significantly more likely among adolescents (male and female) aged 13–21 with behaviorally acquired HIV than among those with perinatally acquired HIV. A 2018 study in England [30] did not find a significant difference in sexual debut between young women and men aged 13–23 with perinatally acquired HIV and those living without HIV. This study found that 28% of the young women in their sample with perinatally acquired HIV had ever had sex, compared to 37% of young women living without HIV.

Although research is limited into why AGYW who acquire HIV before sexual debut are more likely to delay debut and less likely to be currently sexually active than their peers living without HIV or with behaviorally acquired HIV, one hypothesis we have is that at a younger age, knowledge of HIV positive status has more of an impact on sexual risk-taking behavior. Youth who acquire HIV behaviorally have already engaged in sexual risk behavior, by definition, so they would have to change an already established behavior rather than establishing lower risk behaviors at the outset. One study [31] of adolescent girls aged 15–19 living with HIV in Lusaka, Zambia—most of whom reported perinatal HIV infection—found that these adolescents reported high levels of surveillance by their parents and family that discouraged sex/boyfriends and disclosure of status. We recommend further study on the impact of mode of HIV acquisition on sexual behavior. Understanding this relationship will assist with understanding how lived experiences and needed support may differ between AGYW who acquire HIV perinatally and AGYW who acquire HIV behaviorally.

A few other studies have found differences in condom use between those living with and without HIV. The 2018 study in England [30] found that among sexually active adolescents in its sample, those who acquired HIV perinatally were less likely to report that they do not always use condoms (32%), compared to those living without HIV (63%). These proportions are similar to those in our 16–18 age group who reported not always using condoms (instead reporting sometimes or never using them) with serious and casual partners in the last three months. However, the 2014 study in Nigeria found no difference in condom use between sexually active adolescents (male and female) living with and without HIV, with 46.2% of their sample using a condom during their last vaginal intercourse [16]. The 2018 Zambian DHS found current condom use to be 5.4% among sexually active, unmarried women aged 15–19, 1.6% among married women aged 20–24, and 8.7% among sexually active, unmarried women aged 20–24 [24]. These proportions were lower than what we found in our sample.

Although not a comparison between those living with and without HIV, several past studies have shown a reduction in sexual activity and an increase in condom use after participants were notified that they were HIV positive [10–14]. However, none of these studies focused on adolescents, and several of these studies took place outside of SSA and/or focused on men who have sex with men or sex workers, therefore may have limited comparability to our study sample.

We acknowledge that there are limitations to our study. First, participants were asked to recall behaviors over their lifetime and specifically over the last three months, which allows for the potential for recall bias. Due to the stigma surrounding HIV and sexual behavior, especially among those living with HIV, social desirability could have impacted responses. For example, 17.5% of the AGYW reported that they did not know how they acquired HIV. Prior studies have indicated that parents are often reluctant to reveal perinatal infection [32], and participants may also have not been willing to disclose sexually acquired HIV due to stigma. Second, these Data were collected in 2021 during the COVID-19 pandemic, which may have impacted access to services. One review [33] of studies in SSA found an increase in teenage pregnancies and decreased access to sexual and reproductive services during the COVID-19 pandemic. Thus, there may have been reduced access to condoms and other contraceptives during COVID which may be reflected in our study findings. Third, we did not collect information on the HIV status of our participants’ sexual partners, and so were unable to factor this into analysis of condom use. Fourth, our survey did not specifically ask participants whether they were currently having sex, and so our analysis of current sexual activity and current contraceptive use relied on a post hoc definition of current sexual activity based on other survey question responses. Furthermore, we did not have sufficient data on viral load to determine whether those living with HIV in our sample had an undetectable viral load. For those living with HIV who have HIV positive partners or have undetectable viral load, use of condoms may not be needed to prevent the spread of HIV (although condoms would still prevent the spread of other STIs and prevent unwanted pregnancies). Lastly, because the bulk of our analyses was only among participants who reported having had sex, our sample of those living with HIV in the 16–18 age group is relatively small (*n* = 25), which limited statistical power.

Our findings are important for planning tailored HIV interventions based on age and HIV status. We recommend special attention be paid to risk reduction, especially prior to sexual debut. Although we found fewer sexual risk-taking behaviors among those living with HIV, especially in the younger age group, those living with HIV are still in need of tailored interventions. They may also benefit from support in building knowledge and confidence in their ability to engage in safe sex, disclose their status, and build close romantic partnerships, rather than solely focusing on abstinence. Several studies [34, 35] in SSA have looked at the specific sexual health needs of those living with [36–38] and without [39, 40] HIV. Those living without HIV would likely benefit not only from emphasis on the importance of condom use, but also from availability of other options to prevent HIV, such as pre-exposure prophylaxis (PrEP) and HIV testing, which has been found to have low uptake [36, 40].

Our findings also highlight the need for further data on sexual behavior, attitudes toward sex and romantic partnerships, and contraceptive use by HIV status and age to provide insight on how to tailor education content and plan behavioral interventions. AGYW continue to be among the populations at the highest risk of HIV and additional data-driven evidence is needed to develop tailored interventions. These studies should explore in detail issues related to AGYW knowledge, self-efficacy, and access to health care services.

## Conclusion

Young women at risk for HIV require tailored support to increase condom use and risk-reduction behaviors. We found that younger adolescents living without HIV were more likely to have had sex, had younger sexual debut, have more recent partners, and were more likely to be currently having sex than those living with HIV. We found that the young women in the older age group living without HIV were more likely not to use condoms, to be currently having sex, and to have more serious recent partners than their counterparts living with HIV. Therefore, tailored interventions by age group and HIV status are recommended. Those living with HIV may require support to confidently engage in safe sexual relationships. The findings from this study will aid in the design of youth-friendly sexual and reproductive health programs to move towards achieving the Sustainable Development Goal of ensuring access to contraceptive and reproductive services for all people.

## Supplementary Information

Additional file 1. Baseline Survey Questions Used in Analysis. Description of data: Questions in two categories: Sexual behavior and contraceptive use, with response options and skip patterns

## Data Availability

We will be sharing data via a repository identified by the National Institute of Child Health and Human Development. We are planning to post the data later this year (by December 2025) and will provide more information at that time.

## Abbreviations

AGYW: Adolescent girls and young women
ART: Antiretroviral therapy
DHS: Demographic and Health Survey
HIV: Human immunodeficiency virus
PATC^3^H: Prevention and Treatment through a Comprehensive Care Continuum for HIV-Affected Adolescents in Resource Constrained Settings
PrEP: Pre-exposure prophylaxis
SSA: Sub-Saharan Africa
STI: Sexually transmitted infection
