# Supplementary material for: Comparison of sexual risk behaviors among Zambian adolescent girls and young women living with and without HIV
Source: Reprod Health. 2025 Sep 30;22:174. doi: 10.1186/s12978-025-02147-2 (PMC12486779; doi:10.1186/s12978-025-02147-2)
Supplement: Supplementary file 1 — Additional file 1. Baseline Survey Questions Used in Analysis. Description of data: Questions in two categories: Sexual behavior and contraceptive use, with response options and skip patterns [file 12978_2025_2147_MOESM1_ESM.docx]

## Additional File 1: Baseline Survey Questions Used in Analysis

| Survey Question | Response Options^[[1]](#endnote-1),^^[[2]](#endnote-2)^ | Skip Pattern^[[3]](#endnote-3)^ |
| --- | --- | --- |
| Sexual Behavior | | |
| 1. Have you ever had sex? | - Yes - No - Decline to answer |  |
| 2. How old were you when you first had sex? | Text box, integer   - Decline to answer | “Have you ever had sex?” = Yes |
| 3. How many sexual partners have you had in your lifetime? | Text box, integer   - Decline to answer | “Have you ever had sex?” = Yes |
| 4. How many partners in the last 3 months were casual? A casual partner is someone you had sex with occasionally or one time. | Text box, integer   - Decline to answer | “Have you ever had sex?” = Yes |
| 4. How often in the last 3 months did you use condoms with casual partners during sex? | - Never - Less than half the time - About half the time - More than half the time - Always - Decline to answer | “How many partners in the last 3 months were casual?” > 0 |
| 5. How many sex partners in the last 3 months were serious/main partners? A serious partner is someone with whom you've had an ongoing relationship with, like a lover, boyfriend or someone you dated for a while and feel very close to. | Text box, integer   - Decline to answer | “Have you ever had sex?” = Yes |
| 6. How often did you use condoms with serious/main partners in the last 3 months? | - Never - Less than half the time - About half the time - More than half the time - Always - Decline to answer | “How many sex partners in the last 3 months were serious/ main partners?” > 0 |
| Contraceptive Use | | |
| 7. Have you ever used a contraceptive method? This could include condoms, oral contraceptive pill, injections, or implants. | - Yes - No - Decline to answer | “Have you ever had sex?” = Yes |
| 8. What contraceptive methods have you ever used? (Check all that apply.) | - Condoms - Oral contraceptive pill - Injectable - Implant - Other - Decline to answer | “Have you ever used a contraceptive method?” = Yes |
| 9. What contraceptive methods are you currently using? (Check all that apply.) | - None - Condoms - Oral contraceptive pill - Injectable - Implant - Other - Decline to answer | “Have you ever used a contraceptive method?” = Yes |
| 10. What are the reasons that you are not currently using a contraception method? (Check all that apply.) | - I am not currently having sex - I am currently trying to get pregnant - I don't really mind if I get pregnant - Family planning conflicts with my religious beliefs - I don't know what method to use - I don't know where to get family panning - I am worried about how the clinic staff will treat me if I ask for family planning - I am worried about the side effects including infertility - My male partner does not want me to use family planning - My family members do not want me to use family planning - I do not think I can get pregnant - Stories I have heard from people in the community - The cost of family planning - Other reason, specify - Decline to answer | “What contraceptive methods are you currently using?” = None |

1. ⭘ = radio button, can only select one response. [↑](#endnote-ref-1)
2. 🞏 = checkbox, can select multiple options. [↑](#endnote-ref-2)
3. Skip patterns occur when a particular response to one question leads to one or more subsequent questions’ not being asked. [↑](#endnote-ref-3)
